# Synergy between supported ionic liquid-like phases and immobilized palladium N-heterocyclic carbene–phosphine complexes for the Negishi reaction under flow conditions

**DOI:** 10.3762/bjoc.16.159

**Published:** 2020-08-06

**Authors:** Edgar Peris, Raúl Porcar, María Macia, Jesús Alcázar, Eduardo García-Verdugo, Santiago V Luis

**Affiliations:** 1 Dpt. of Inorganic and Organic Chemistry, Supramolecular and Sustainable Chemistry Group, University Jaume I, Avda Sos Baynat s/n, E-12071-Castellon, Spain; 2 Discovery Chemistry, Janssen Research and Development, Janssen-Cilag, S.A., C/ Jarama 75A, Toledo, Spain

**Keywords:** immobilized catalyst, Negishi cross-coupling, NHC complex, palladium, supported ionic liquid

## Abstract

The combination of supported ionic liquids and immobilized NHC–Pd–RuPhos led to active and more stable systems for the Negishi reaction under continuous flow conditions than those solely based on NHC–Pd–RuPhos. The fine tuning of the NHC–Pd catalyst and the SILLPs is a key factor for the optimization of the release and catch mechanism leading to a catalytic system easily recoverable and reusable for a large number of catalytic cycles enhancing the long-term catalytic performance.

## Introduction

N-heterocyclic carbenes (NHCs) are known as efficient coordination ligands for different types of metals. The main feature of NHC complexes is their structural tunability [1]. Thus, their catalytic efficiency can be easily modulated through systematic variations of the steric and electronic design vectors of the NHC ligand [2]. These complexes have been used as highly efficient catalysts for a wide variety of C–C and C–X cross-coupling reactions [3]. Among others, different NHC–Pd complexes have been designed as efficient homogeneous catalysts for Negishi reactions [4]. Although these systems are highly efficient, their homogeneous nature hamper the separation of the products and recovery of the excess of the palladium from the reaction solution. A possible solution to this issue is the preparation of the related immobilized complexes enabling a simpler recovery and reuse of the catalysts by filtration [5]. Furthermore, the immobilized NHC-complexes can be easily adapted to flow processes using a fix-bed reactor set-up increasing simultaneously the sustainability and the efficiency of the C–C coupling reactions [6–7]. In the pursuit of NHC immobilized metal complexes many different materials of organic and inorganic nature have been used as supports [5]. Several reports describe the synthesis of supported palladium–NHC complexes (Pd–NHC) and their application in cross-coupling reactions [8–10]. The main flaw of this type of systems is related with the mechanistic aspect of most C–C formation reactions catalyzed by palladium [11]. Careful studies of the reaction mechanism have revealed that, under certain conditions, palladium-supported species can act as mere pre-catalysts [12–13]. As suggested by Ananikov and co-worker, these in situ-generated catalytic species can give rise to cocktail-type systems with different active metal species present in solution [14]. In the case of immobilized catalysts, these palladium species can be released from the support reacting in the homogeneous medium transformed from Pd^0^ to Pd^II^. In this way, a “cocktail” of catalytic species, including molecular complexes, clusters and nanoparticles may be responsible for the catalytic activity [15]. In a continuous system, this can be accompanied by a significant leaching of the catalytic metal. In order to avoid a loss of activity by this leaching process, the role of the ligand and the support on the evolution of the catalytic systems should be carefully considered. It has been shown that the immobilization of Pd-catalysts or precatalysts onto supported ionic liquid-like materials can facilitate the recapture by the support of the released active species at the end of the reaction, in what has been called a “boomerang” mechanism [16–17]. Here, we report our efforts aiming a rational development for Negishi catalysts based on NHC–Pd complexes in conjunction with supported ionic liquid-like phases to enhance their catalytic stability under continuous flow conditions.

## Results and Discussion

**Scheme 1:**
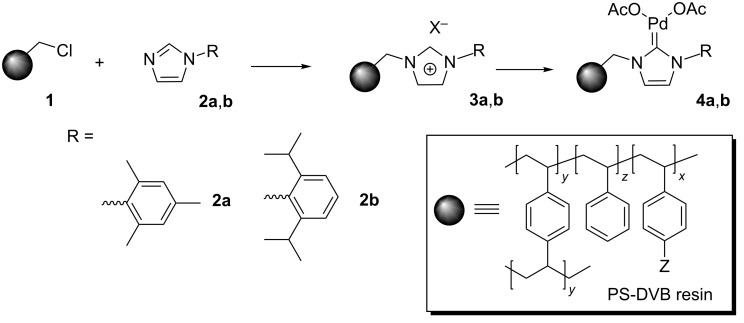
Synthesis of NHC-supported catalysts.

Scheme 1 summarizes the synthetic approach used for the preparation of the functionalized polymers considered in this work. A commercially available PS-DVB bead-type macroporous chloromethylated polymer **1** (Merrifield resin with 20% DVB and 1.2 mmol Cl/g,) was used as the starting material for the immobilization of *N*-arylimidazoles **2a** and **2b**, following the traditional alkylation protocol [18–19]. The preparation of these modified polymers was monitored by FT–ATR–IR, FT-Raman using a micro-spectroscopy accessory and the NBP test [20–21]. The corresponding Pd–NHC complexes **4a** and **4b** (Scheme 1) were obtained by treatment of the supported imidazolium species with Pd(OAc)_2_ in the presence of a base. The amount of immobilized NHC ligand was determined by elemental analysis, while the palladium loaded on the polymer was determined by ICP analyses (see Table 1). The slightly lower value of the Pd loading is likely to be related to the partial formation of 2:1 NHC/Pd complexes. The experimental details for the synthesis of **2a**,**b**, **3a**,**b** and **4a**,**b** are given in Supporting Information File 1.

**Table 1:** Pd Loading for the different NHC-Pd synthesized.

Entry	Polymer	NHC loading^a^	Pd loading^b^

1	**4a**	0.75	0.43
2	**4b**	0.64	0.46

^a^As determined by elemental analysis (mmol functional group/g resin); ^b^as determined by ICP analysis (mmol Pd/g resin).

**Scheme 2:**
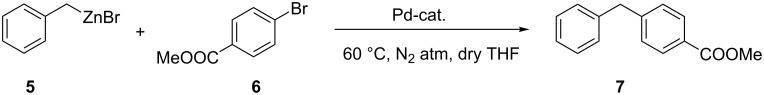
Negishi benchmark reaction.

The Negishi reaction of benzylzinc bromide (**5**) and methyl 4-bromobenzoate (**6**) to form methyl 4-benzylbenzoate (**7**) was used to evaluate the activity of the palladium catalysts on polymer support (Scheme 2). The Negishi reaction is a potent cross-coupling reaction in organic chemistry. Notably, it has much value for the synthesis of fine chemicals and medicinal drugs [22]. The conditions selected for the benchmark reaction were the use of THF as the solvent, 60 °C and a catalyst loading based on the introduction of 5 mol % of Pd. The reaction was monitored by GC and the resulting conversions and yields were confirmed by ^1^H NMR (see Supporting Information File 1).

**Figure 1:**
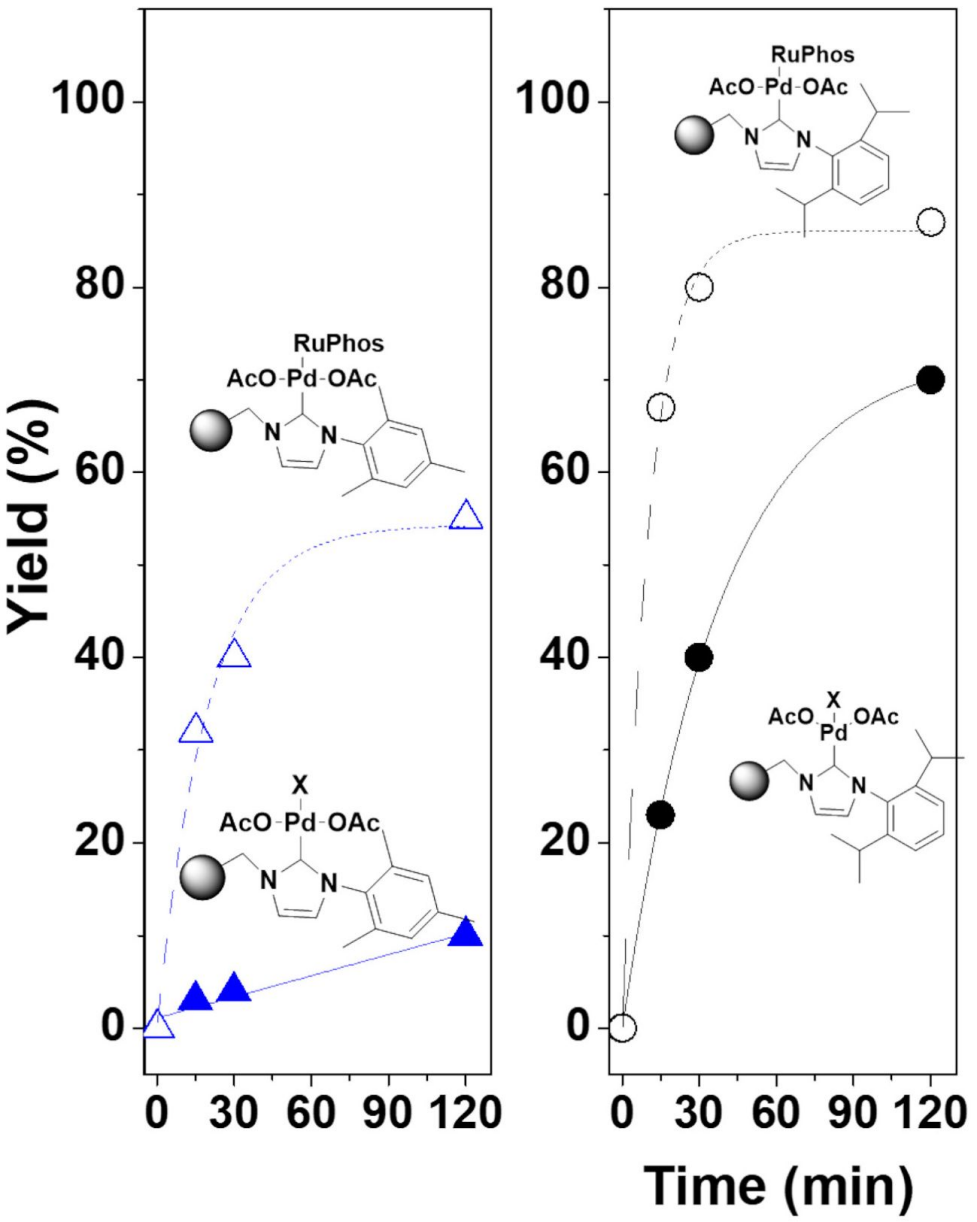
Negishi reaction catalyzed by immobilized NHC–Pd complexes. Conditions: methyl 4-bromobenzoate (0.25 mmol, 1 equiv), benzylzinc bromide (0.5 mmol, 2 equiv, 1 mL of a 0.5 M solution in THF), 5 mol % of Pd catalyst (0.0125 mmol) in dry THF (1 mL). N_2_ atmosphere, 60 °C. Yields calculated by GC and confirmed by ^1^H NMR.

The kinetic plots for this model reaction are represented in Figure 1. The NHC–Pd catalyst **4a** showed a rather reduced activity (less than 10% after two hours), while the catalyst bearing isopropyl moieties at the aromatic ring (**4b**) displayed a significant increase in the catalytic activity, reaching 67% yield after 120 min (Figure 1).

**Scheme 3:**
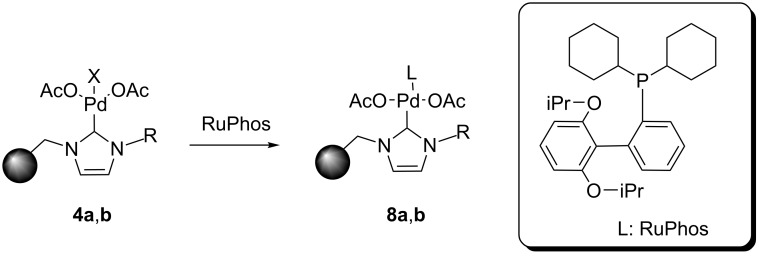
Synthesis of immobilized NHC–Pd–RuPhos.

In order to improve the catalytic performance of these systems, the cooperative effect of an additional ligand was evaluated. Organ and co-workers have demonstrated that the introduction of pyridine ligands can be used to enhance the activity of such NHC complexes (pyridine enhanced precatalyst preparation, stabilization and initiation, PEPPSI) [23–24]. In addition to pyridine ligands, other compounds with coordinating atoms such as C, N or P have been reported to tune the catalytic activity of the NHC–Pd complexes [25–28]. Thus, a ligand containing P as the coordinating atom was selected for the activation of the NHC complexes. In this regard, RuPhos (2-dicyclohexylphosphino-2′,6′-diisopropoxybiphenyl) has been used as a palladium ligand for the Negishi reaction [29]. The preparation of the NHC–Pd–RuPhos complexes **8a**,**b** was carried out by mixing a suspension of **4a**,**b** with a solution of RuPhos for 2 hours (Scheme 3). The corresponding modified immobilized NHC–Pd–RuPhos complexes were isolated by filtration and thoroughly washed to remove any remaining non-coordinated RuPhos (0.37 and 0.57 mequiv of Pd/g for **8a** and **8b**, respectively).

The introduction of the additional phosphine ligand produced a clear positive effect on the activity, enhancing the catalytic performance of the immobilized NHC–Pd complexes assayed as clearly shown in the kinetics profiles depicted in Figure 1. Both NHC–Pd–RuPhos catalysts showed an activity increase: ca. 10-fold for **8a** and ca. 2.9-fold for **8b**, according to the TOF values calculated at 15 minutes.

**Figure 2:**
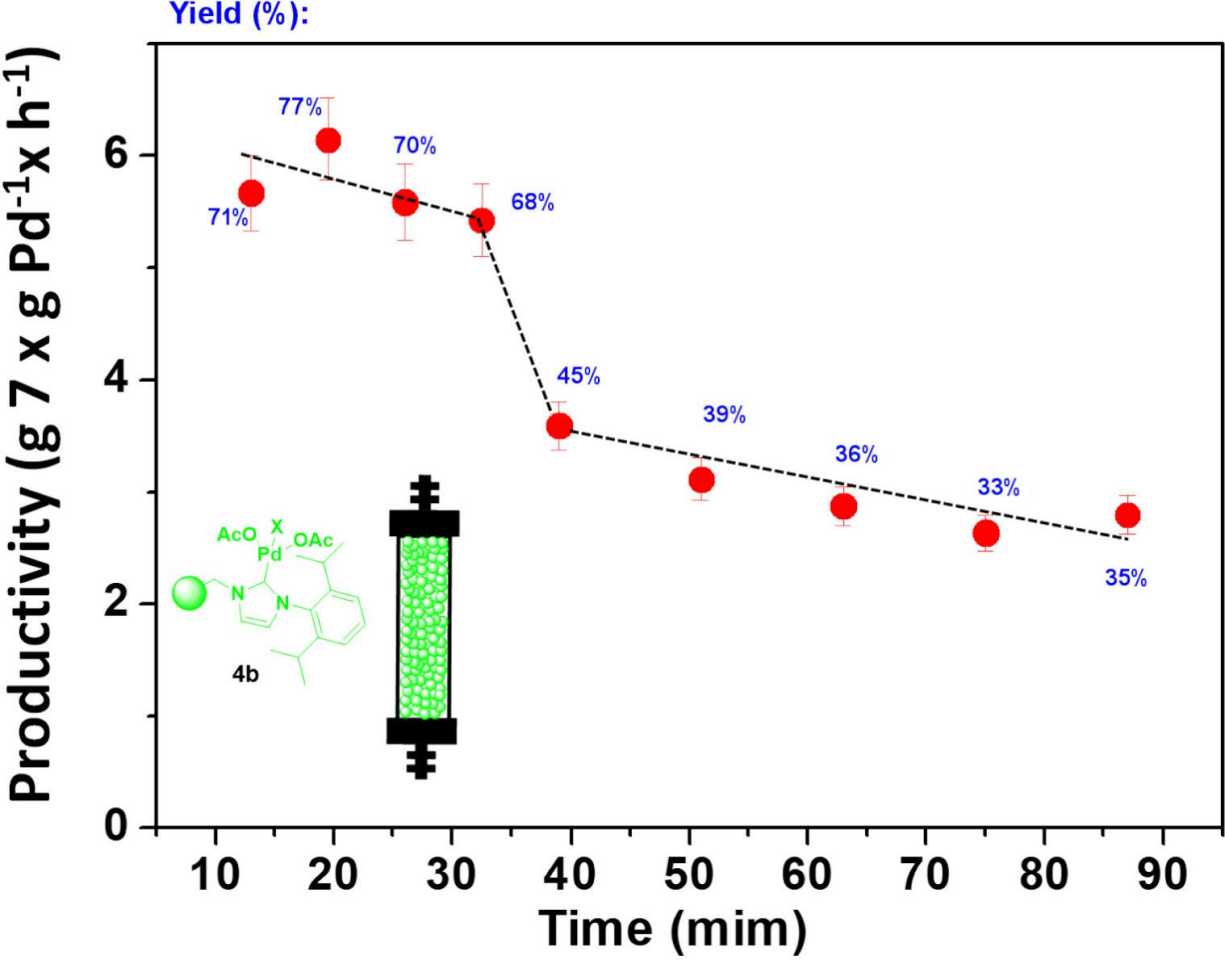
Negishi model reaction between **5** and **6** under flow conditions catalyzed by **4b**. *V* = 0.535 mL, 363 mg of **4b**, residence time = 2.5 min, total flow rate 0.214 mL/min. Productivity max: 7.97 g of **7** × g Pd^−1^ × h^−1^. Yields calculated by GC and confirmed by ^1^H NMR.

In the light of this initial screening under batch conditions with both kinds of catalytic complexes, NHC–Pd (**4a**,**b**) and NHC–Pd–RuPhos (**8a**,**b**), their activity and stability was evaluated under flow conditions. For this, the corresponding fixed-bed reactors were prepared, and the general flow reaction setup depicted in Supporting Information File 1. It should be mentioned that the catalyst **4a** showed, as in the batch process, low activity (<5% yield) under flow conditions. However, the catalyst **4b** yielded **7** under the selected flow conditions (flow rate of 0.214 mL/min, Figure 2). At initial times, yields for **7** were higher than those obtained in the batch process, which is remarkable considering the short residence time used (2.5 min). However, a strong deactivation was observed under prolonged use (Figure 2). The catalytic activity decay calculated in terms of productivity (g of **7** × g Pd^−1^ × h^−1^) was of ca. 50% after only 2 h of continuous use. This decay in activity can be associated with the leaching of palladium from the heterogeneous phase [30]. The initial samples collected showed an elevated concentration of leached soluble palladium species as calculated by ICP–MS of the respective solutions (>15 ppm of Pd). The elemental analysis of the catalyst after its use was also consistent with this Pd loss from the NHC complex.

**Figure 3:**
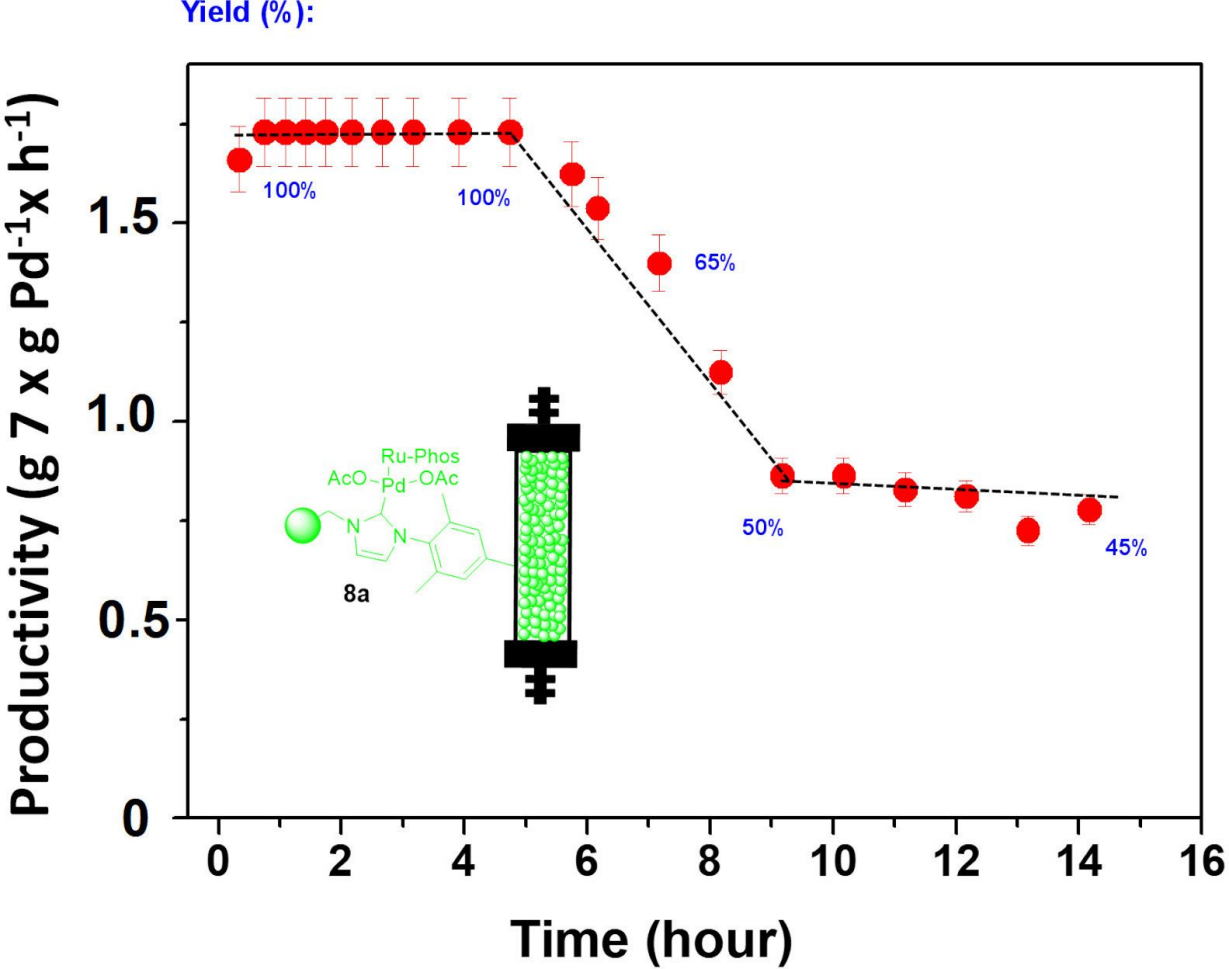
Negishi model reaction under flow conditions catalyzed by **8a**. *V* = 2.9 mL, 1.25 g of catalyst, residence time = 29 min, total flow rate: 1 mL/min, productivity max: 1.73 g of **7** × g Pd^−1^ × h^−1^. Yields calculated by GC and confirmed by ^1^H NMR.

Additionally, the catalyst **8a** was also tested under continuous flow conditions. Although, this time a fivefold larger fixed bed reactor (2.9 mL vs 0.5 mL) was used, leading to a larger residence time (29 min vs 2.5 min). An increase in the residence time may favor the re-adsorption of the active species into the support limiting, at some extent, the Pd leaching. The results obtained under these conditions are depicted in Figure 3. As expected, the use of a larger residence time led to a high yield. Quantitative yields were initially obtained under these conditions, with a productivity of 1.73 g **7**/g Pd·h. This level of activity was kept constant during at least 5.5 h of continuous use. However, after this time a strong deactivation of the catalyst took place. An activity loss of ca. 50% of the initial value was observed. After this decay, between 8 and 15 h of continuous use, the productivity achieved was maintained around 0.8 g of **7** × g Pd^−1^ × h^−1^.

These results agree with the ones observed by Organ and co-workers, who have developed silica-immobilized Pd–PEPPSI–*IPr*–SiO_2_ [31] and Pd–PEPPSI–*IPent*–SiO_2_ [32] catalysts. They observed a gradual catalyst deactivation due to the slow release of palladium over time. However, the high level of catalyst activity, especially for Pd–PEPPSI–*IPent*–SiO_2_, made, in that case, the loss of Pd less relevant. With a reduction of palladium loading to half of its initial value, still relatively efficient long-term flow runs could be carried out.

**Figure 4:**
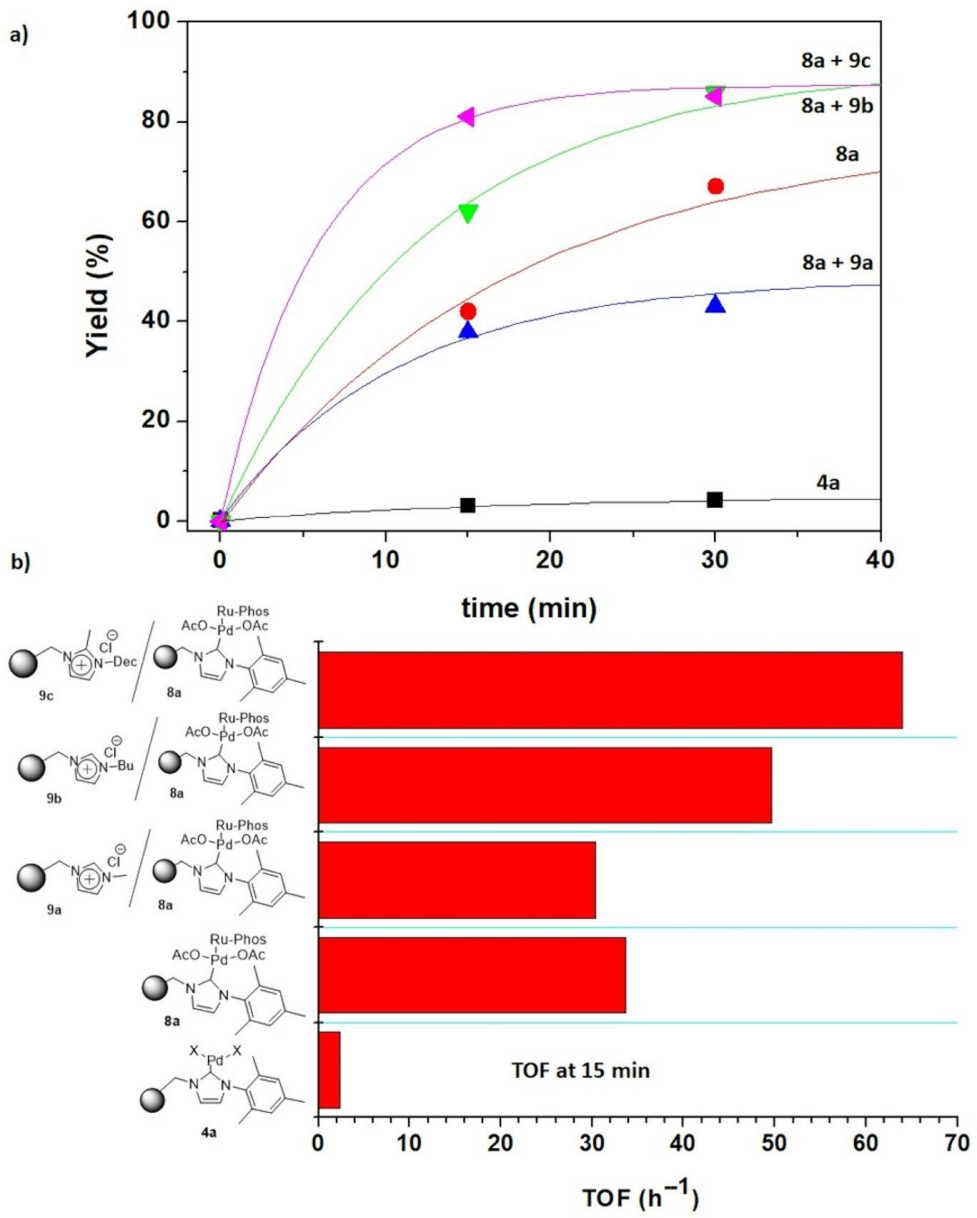
Negishi reaction between **5** and **6** catalyzed by **8a** in the presence of SILLPs**.** a) Yield (%) vs time for different catalytic mixtures. b) TOF values obtained at 15 minutes. Conditions: 1 equiv methyl 4-bromobenzoate (0.25 mmol), 2 equiv benzylzinc bromide (0.5 mmol, 1 mL of a 0.5 M solution in THF), 5 mol % Pd catalyst (0.0125 mmol) in dry THF (1 mL). N_2_ atmosphere. Yields calculated by GC and confirmed by ^1^H NMR.

Different strategies have been evaluated to develop precatalysts/catalysts immobilized onto supported ionic liquid-like phases (SILLPs) [33–38]. In these systems, the microenvironment provided by the ionic liquid-like units can have a remarkable influence on the overall process, particularly on the catalytic activity and recyclability of the supported species. Indeed, the appropriate design of the SILLPs is a key factor for the optimization of release and catch systems leading to easily recoverable and reusable catalysts working for a large number of catalytic cycles without any loss in performance [33–34]. In this regard, the effect of the presence of different SILLPs in the catalytic behavior of the former catalytic complexes was evaluated. Thus, the benchmark Negishi reaction between **5** and **6** was performed using a polymeric mixture of two components: the immobilized NHC–Pd–RuPhos catalyst and a series of polymeric SILLPs in a 1:3 weight ratio. The first component can efficiently act as a catalyst but also will generate and release a series of Pd species accounting for the leaching. The second component, the SILLP, can act as scavenger of those species leached to the solution not only eliminating them from the solution but also contributing to their stabilization avoiding the formation of inactive species and keeping their activity for further catalytic cycles. Three different SILLPs were evaluated displaying different imidazolium substitution patterns and a loading of IL-like fragments of 13–24% by weight. Figure 4 summarizes the results obtained for this study. The presence of SILLPs affected both activity and Pd leaching. Regarding the activity, the presence of methyl imidazolium groups in SILLP **9a** slightly reduced the catalytic activity, while the presence of butyl- and methyldecylimidazolium units (SILLPs **9b** and **9c**, respectively) led to more active systems. This can be clearly appreciated when the TOF values at 15 minutes are compared (Figure 4b). The presence of the SILLPs **9c** and **9b** clearly led to a catalytic system more active than **8a**, while the presence of **9a** reduced the activity. The presence of the SILLPs also had a strong influence on the Pd leaching. In the absence of the SILLPs (**8a** as catalyst) the observed Pd leaching was 22.4 ppm, corresponding to ca. 7% of the initial Pd. In the presence of the SILLPs this leaching was reduced to 2.7, 14.4 and 17.1 ppm (ca. 0.8%, 4.5% and 5% of the initial Pd) for the polymeric mixtures **8a** + **9a**, **8a** + **9b** and **8a** + **9c**, respectively. This confirmed that the SILLPs act as scavengers for the leached Pd species. Noteworthy, the larger palladium leaching did not lead to higher activities, suggesting that in absence of the SILLP some deactivation for the leached species was taking place.

**Figure 5:**
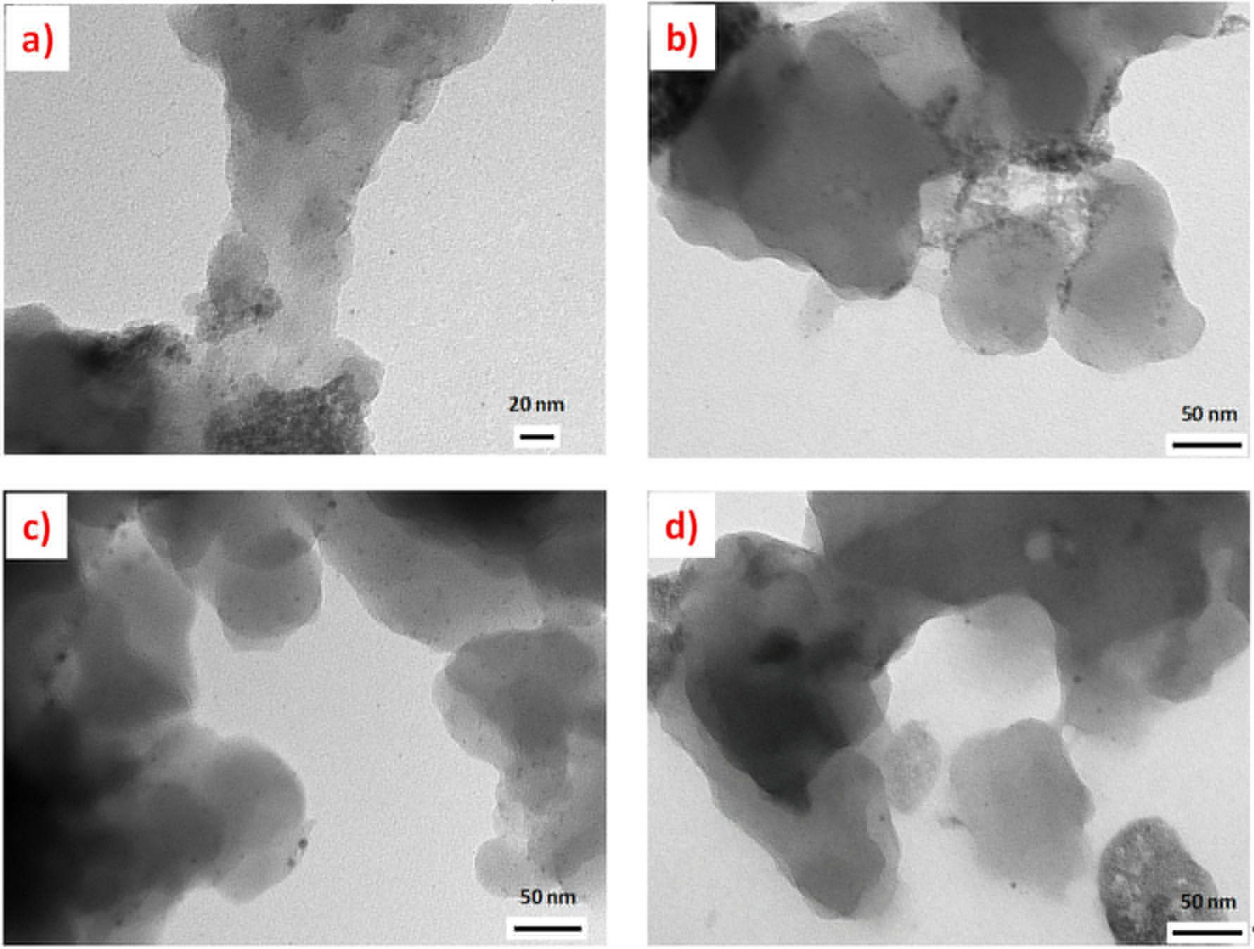
TEM images of the polymers after the Negishi reaction between **5** and **6**. a) **8a**, bar scale 20 nm, PdNP size distribution 4.91 ± 1.26 nm. b) **8a** + **9a**, bar scale 50 nm, PdNP size distribution 4.23 ± 1.65 nm. c) **8a** + **9b**, bar scale 50 nm, PdNP size distribution 3.61 ± 1.36 nm. d) **8a** + **9c**, bar scale 50 nm, PdNP size distribution 3.23 ± 0.81 nm.

The TEM analysis of the polymers after the reactions showed the presence of palladium nanoparticles (Figure 5) confirming that during the reaction a part of the leached palladium was converted into PdNPs. The higher reactivity of the cocktails based on **9b** and **9c** can be associated with the presence of PdNPs with smaller size distributions. Our previous results obtained for AuNPs-SILLPs indicated that for the macroporous resins the NP size decreased when the size of the aliphatic residue of the imidazolium units increased with decyl N-substitution leading to smaller particle sizes [39]. The analysis of the polymeric samples revealed similar trends for the case of PdNPs. SILLPs with decyl and butyl N-substitution (**9c** and **9b**) presented the smallest size distributions, being also the most reactive ones.

**Scheme 4:**
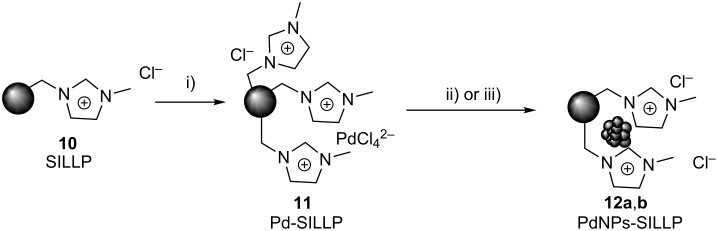
Pd species immobilized onto SILLPs. i) 1 g SILLP **10,** 100 mg PdCl_2_ in milli-Q^®^ water (100 mL 1% HCl, 1000 ppm PdCl_2_), orbitalic stirring, rt, 5 h. ii) 250 mg Pd-SILLP **11**, 0.2 g NaBH_4_ in 12 mL EtOH/H_2_O 1:4, rt, 3 h. iii) 250 mg Pd-SILLP **11**, 4 mL EtOH, MW (2 h, 200 °C, 300 psi, 120 W).

In the light of these experiments, it can be concluded that the supported NHC–Pd–RuPhos **8a** acts as both catalyst and as a system releasing soluble Pd species, which can be partially catched by the SILLP acting as scavenger. The key is to find out if these recaptured species onto SILLP are still active for the Negishi reaction. The leaching of Pd from NHC–Pd–RuPhos is in agreement with previous studies for C–C palladium catalyzed reactions [33–35]. Recently, Ananikov and co-workers reported that for Pd–NHC systems the reactivity of the systems is mainly determined by the cleavage of the metal−NHC bond, while the catalyst performance is strongly affected by the stabilization of in situ-formed metal clusters [15,30,40]. In the mechanism suggested by these authors, Pd–NHC complexes can evolve through two different pathways towards the formation of a catalytically active cocktail of Pd species. In the first one, a reductive elimination takes place from the Pd(II) intermediate with the concomitant release of NHC-containing byproducts. In the second pathway, the dissociation of the M–NHC produces Pd intermediates from which metal clusters and MNPs can be readily formed while the carbene can react through C−NHC or H−NHC coupling. The presence of onium salts significantly contributes to the stabilization of both Pd clusters and PdNPs [40]. Thus, a combination of a classical molecular mode of operation and a cocktail-type mode of operation can also be involved in the Negishi reaction. Two different palladium species released from the NHC complex can act as catalyst for the Negishi reaction: i) soluble Pd(II) species or ii) palladium nanoparticles (PdNPs). Imidazolium moieties in SILLPs can scavenge and stabilize both types of palladium species. The possible catalytic effect of these Pd species immobilized onto SILLPs for the Negishi reaction was, thus, evaluated. Firstly, a solution of Pd(II) was adsorbed in the SILLP **10** similar to **9a** but with a high loading of methylimidazolium units leading to a Pd(II)-SILLP system **11** with 0.56 mequiv of Pd/g of SILLP and 3.79 mequiv of IL-like units/g of SILLP (Scheme 4). This system was treated with either NaBH_4_ or EtOH under microwave irradiation to produce the corresponding PdNPs immobilized onto SILLPs (**12a**,**b**).

**Figure 6:**
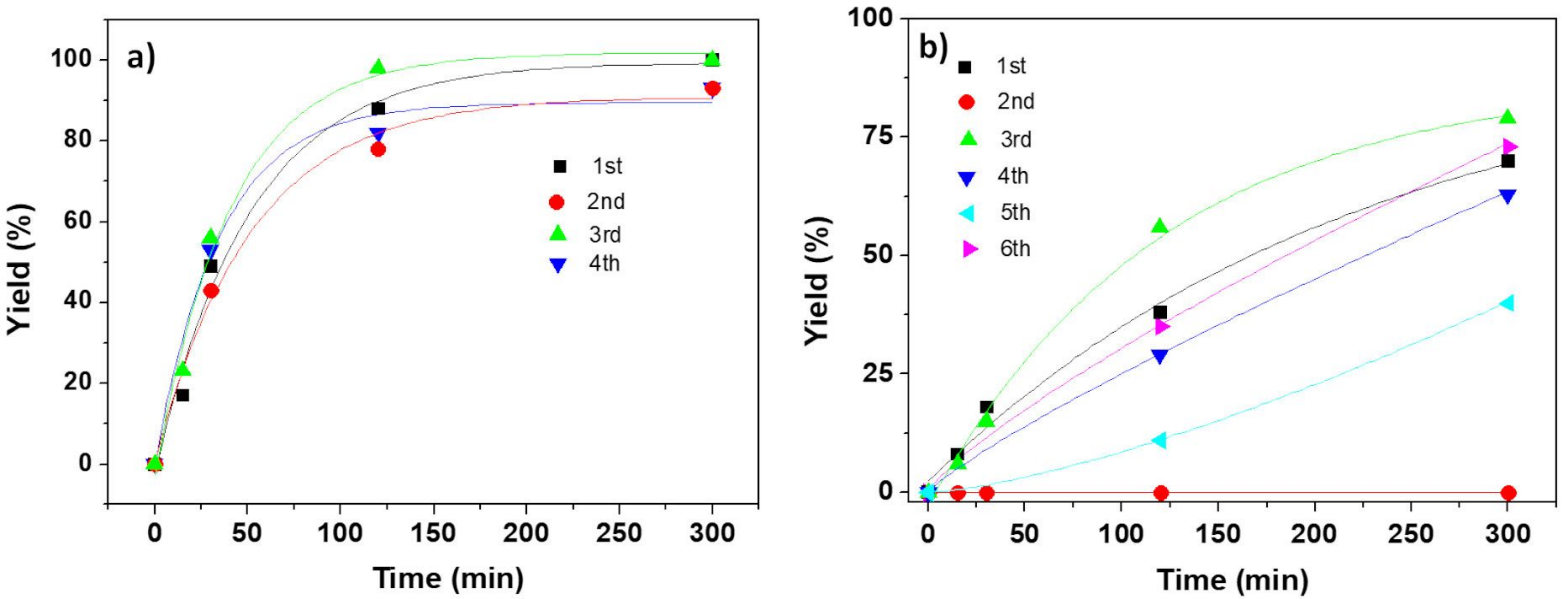
Negishi reaction between **5** and **6** catalyzed by **11**. 1 equiv methyl 4-bromobenzoate (**6**, 0.25 mmol), 2 equiv benzylzinc bromide (**5**, 0.5 mmol, 1 mL of a 0.5 M solution in THF), 5 mol % Pd catalyst in dry THF (1 mL). a) **11** in presence of RuPhos, Pd/RuPhos 1:0.05 molar ratio. RuPhos added for each cycle. b) **11** + **9a**, 1:3 weight ratio, in the presence of RuPhos, Pd/RuPhos 1:1 molar ratio, RuPhos added each cycle except for the 2nd cycle. N_2_ atmosphere. Yields calculated by GC and confirmed by ^1^H NMR.

The Pd-containing polymers **11** and **12a**,**b** were tested as potential catalysts for the benchmark Negishi reaction. Pd(II) adsorbed by ionic exchange (**11**) yielded 73% of **7** after 120 minutes of reaction under standard conditions, confirming that soluble Pd(II) species released from the immobilized systems and scavenged by SILLPs can act as catalysts for the Negishi reaction. The reaction was also evaluated in the presence of 0.05 equivalents of RuPhos, as some of this ligand should be released from the NHC–Pd–RuPhos complex along with Pd (Figure 6a). Under such conditions, the reaction took place with yields for **7** of ca. 90% for the first cycle. Noteworthy the levels of leaching were lower than the ones observed for **8a** (0.49 ppm vs 22.4 ppm for **8a**). The reaction was also assayed in the presence of one equivalent of RuPhos and SILLP **9a** as scavenger, by using a mixture of **11** and **9a** in a 1:3 weight ratio (Figure 6b). Under these conditions, the catalytic system was less active, but the leaching was reduced even further reaching a value of 0.04 ppm. The recyclability of the systems was also tested. In general, the catalysts assayed maintained the catalytic activity as far as an additional amount of RuPhos (0.05 equiv) was added for the new cycle. Under these conditions, the activity of **11** was kept constant for at least four catalytic cycles remaining the Pd leaching for each cycle rather small (ca. 0.04 ppm in the solution). The mixture of **11** and the scavenger **9a** was also active in successive cycles, under similar conditions, although the activity suffered from more fluctuations, probably due to the heterogeneity of the mixture. In any case, the systems were still active after six consecutive cycles being the Pd leaching per cycle in the 0.04–0.12 ppm range. It can be seen that in the absence of an additional amount of RuPhos after the first cycle the catalytic activity was lost but was recovered for the third cycle when RuPhos was added.

Regarding the activity of the PdNP-SILLPs **12a**,**b**, they showed no activity, with yields lower than 1%, in the absence of RuPhos, while providing good catalytic performance in the presence of one equivalent of the phosphine. The catalysts prepared by NaBH_4_ reduction were slightly less reactive than those obtained with EtOH as reducing agent. Noteworthy, the supported catalysts were active in further catalytic cycles after separation of the product by filtration and polymer washing [41]. Catalytic systems **12a**,**b** were also recycled being **12a** even more active in a second than in the first cycle while **12b** reduced its activity (see Table 2). To understand these differences, it must be noted that the nature of the MNPs obtained in SILLPs is very sensitive to the procedure used for their preparation and this significantly affects its activity but also their recyclability [35,39]. The capacity to generate active catalytic species from the MNPs is essential in the first run, but the capacity of the system, including imidazolium fragments and remaining MNPs, to efficiently recapture the soluble species in an active form is key for the second and successive runs.

**Table 2:** Negishi reaction between **5** and **6** catalyzed by **12a**,**b**.^a^

Entry	Catalyst	Pd/RuPhos	Cycle	Yield (time, min)^b^		

				15	30	120

1	**12a**	1:0	1	n.f.	n.f.	n.f.
2	**12b**	1:0	1	n.f.	n.f.	n.f.
3	**12a**	1:1	1	12	31	73
4	**12b**	1:1	1	42	50	73
5	**12a**	1:1	2	84	83	85
6	**12b**	1:1	2	11	21	66

^a^1 equiv methyl 4-bromobenzoate (**6**, 0.25 mmol), 2 equiv benzylzinc bromide (**5**, 0.5 mmol, 1 mL of a 0.5 M solution in THF), 5 mol % Pd catalyst in dry THF (1 mL). ^b^Yield calculated by GC and confirmed by ^1^H NMR; n.f.: product not found.

**Figure 7:**
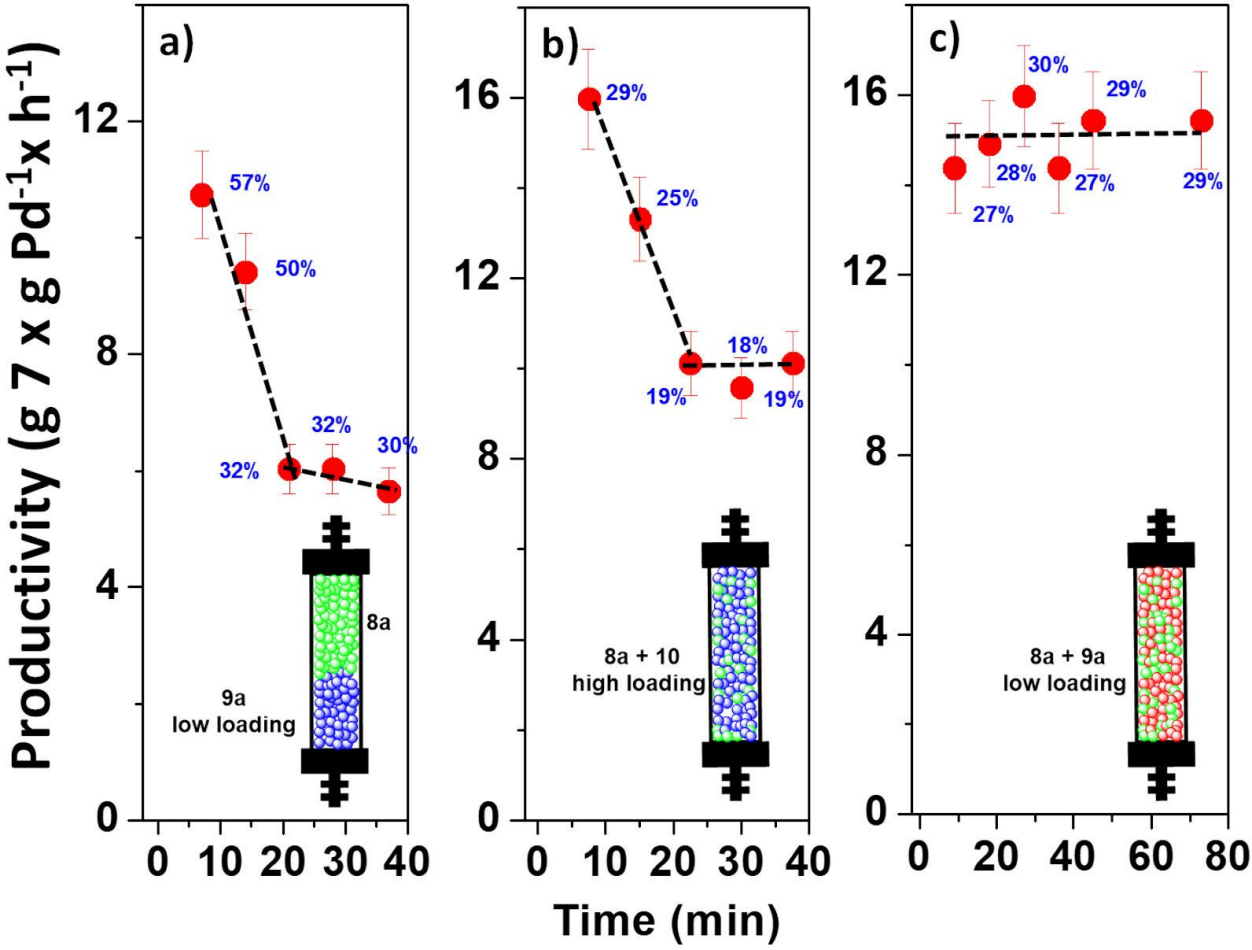
Negishi reaction between **5** and **6** under flow conditions catalyzed by **8a** in the presence of a scavenger SILLP (**9a** and **10**). a) 200 mg of **8a** (top) and 200 mg of **9a** (bottom). *V* = 0.535 mL, residence time = 2.5 min, productivity max: 18.84 g **7** × g Pd^−1^ × h^−1^. b) 50 mg of **8a** and 150 mg of **10** (mixed), *V* = 0.38 mL, residence time = 2.5 min, Productivity max: 53.24 g **7** × g Pd^−1^ × h^−1^. c) 50 mg of **8a** and 150 mg of **9a** (mixed), *V* = 0.35 mL, residence time = 2.5 min, productivity max: 53.24 g **7** × g Pd^−1^ × h^−1^. Yield calculated by GC and confirmed by ^1^H NMR.

All these results suggest that the SILLPs can be used as efficient scavengers for the palladium-leached species released from NHC–Pd–RuPhos complexes, limiting the leaching and possibly improving the long-term system stability. In order to screen the effect of the SILLPs under continuous flow conditions, small flow fixed-bed reactors were prepared and evaluated. The first system was prepared by packing two layers, one on the top of the other. The bottom layer was prepared with 200 mg of the scavenger SILLP **9a** (to recapture released Pd species) and the top layer with 200 mg of the catalyst **8a** (Figure 7a). However, this configuration did not contribute to improve the stability of the system, with a strong catalyst deactivation observed, reaching ca. 47% of the initial activity after 40 h on flow.

In an attempt to achieve a better performance a homogeneous distribution of the catalyst and the scavenger within the fixed bed reactor seems to be preferable as it has been observed in multicatalytic systems [42]. In this case, two different fixed bed reactors were prepared with a well-disperse mixture formed by 50 mg of the catalyst **8a** and 150 mg of a SILLP. Two different SILLPs were used, one with low loading of IL-like units (**9a**,1.09 mequiv/g, 13 wt %) and one with a high loading (**10**, 3.79 mequiv/g, 37 wt %). In the case of the continuous reactor containing the high loading SILLP the activity decay was lower than the previously observed (up to ca. 35% of the initial value). However, the system based on the use of the SILLP **9a** with a low loading kept a constant level of activity, with a productivity of ca. 15 g of **7** × g Pd^−1^ × h^−1^.

**Figure 8:**
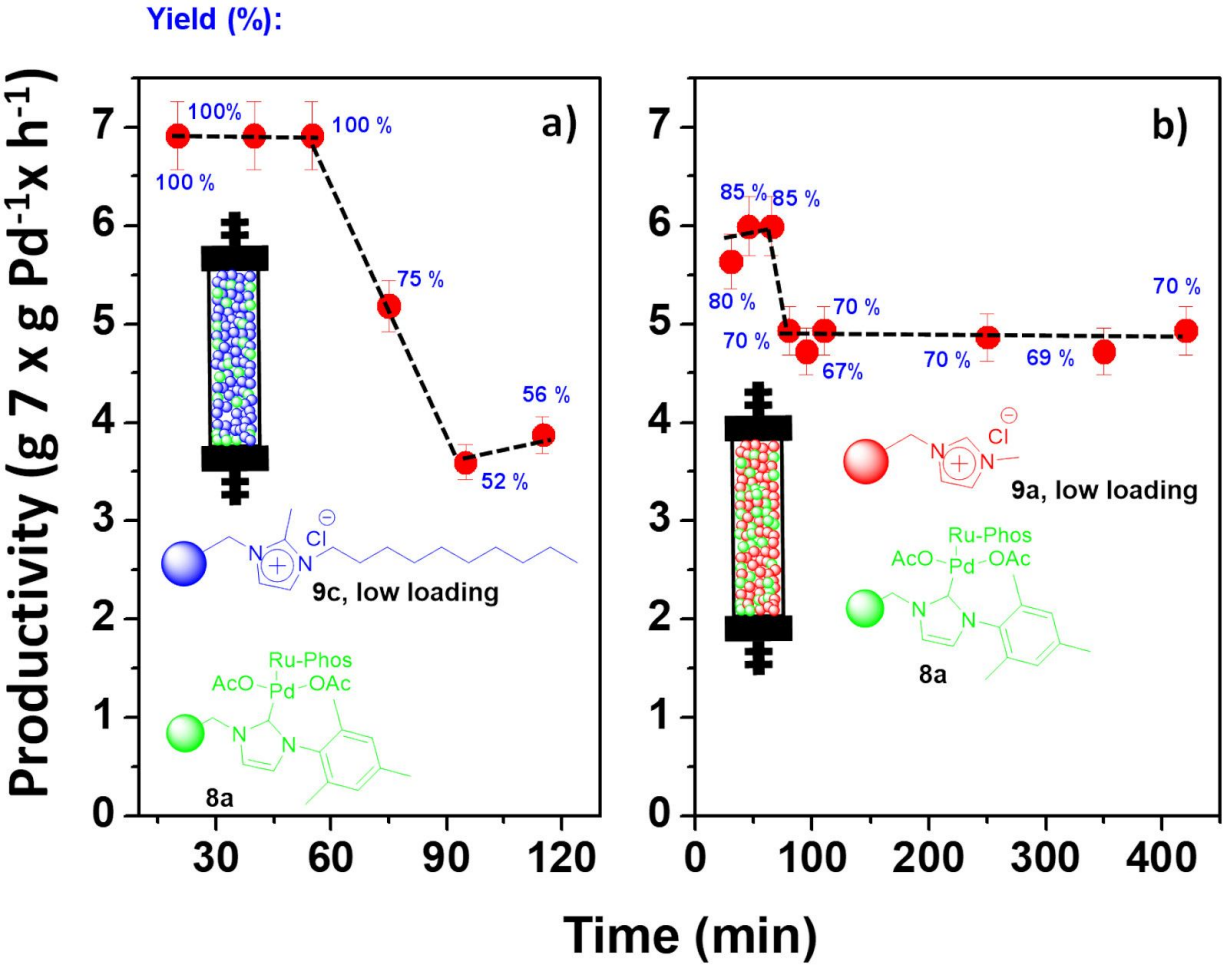
Effect of the structure of the SILLP scavenger for the Negishi reaction between **5** and **6** under flow conditions catalyzed by **8a** in the presence of SILLPs **9a** or **9c**. 60 °C. Total flow rate: 0.1 mL/min. 0.05 mL of a 0.2 M solution of **5** in THF and 0.05 mL/min of a 0.1 M solution of **6** in THF. *V* = 1.7 mL, residence time = 17 min. Productivity max: 7.08 g **7** × g Pd^−1^ × h^−1^. a) 250 mg of **8a** and 750 mg of **9c** SILLP low loading, b) 250 mg of **8a** and 750 mg of **9a** SILLP low loading. Yield calculated by GC and confirmed by ^1^H NMR.

Based on these results, two larger fixed bed reactors were prepared, and their performance evaluated for the benchmark Negishi reaction. A first one was filled with 250 mg of **8a** and 750 mg of the SILLP **9a**, while the second one contained the same amount of catalyst but using the SILLP **9c** instead of **9a**. The results are summarized in Figure 8. In agreement with results observed in the batch experiments, the mixture of the polymers **8a** + **9c** led to more active systems reaching >99% yield of **7**. However, the activity strongly decayed after 60 minutes of continuous use. This can be related with the leaching observed during this period with samples containing up to 10 ppm of leached palladium. The system based on **8a** and **9a** provided a more stable performance. Although the activity displayed was slightly lower, as observed in the batch experiments, it remained constant after an initial conditioning time. Thus, the initial samples showed up to ca. 85% yield of **7**, which after 80 minutes slightly decayed to ca. 70% yield, corresponding to a productivity of 4.87 g of **7** × g Pd^−1^ × h^−1^. This level of productivity was maintained during at least five hours of continuous use.

**Figure 9:**
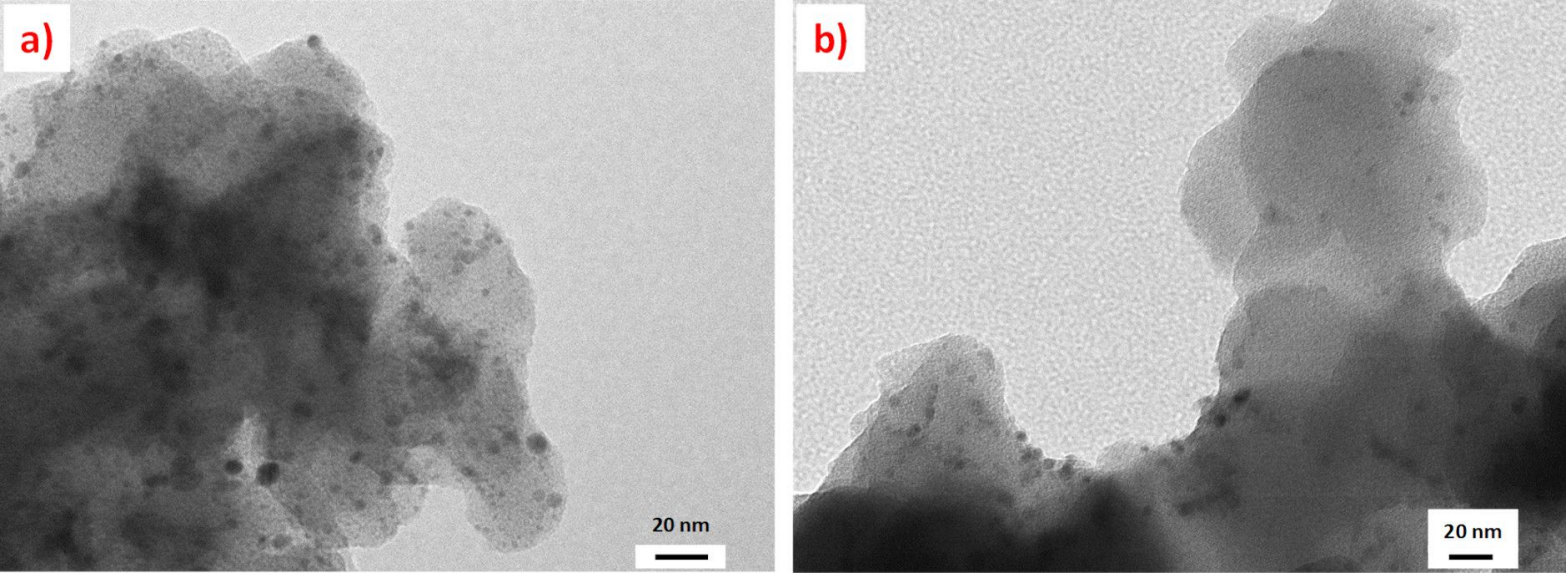
TEM images of the polymer after the Negishi reaction between **5** and **6** under flow conditions. a) **8a** + **9c**, bar scale 20 nm, PdNPs particle size distribution 4.72 ± 1.44 nm. b) **8a** + **9a** bar scale 20 nm, PdNPs particle size distribution 3.12 ± 0.97 nm.

The TEM images of the polymeric systems after their use under continuous flow conditions revealed again the presence of PdNPs (Figure 9). However, the images show that for the system **8a** + **9c** the number of particles and their size distribution are larger than for **8a** + **9a**. This trend is different to the one observed in batch experiments.

## Conclusion

The results here presented confirm the viability of using polymeric cocktails formed by mixtures of supported NHC–Pd–RuPhos and SILLPs as efficient catalysts for the Neghishi reaction. In such cocktails, SILLPs act as scavengers of the palladium species released from the immobilized NHC–Pd–RuPhos, leading to complex mixtures of immobilized species still active for the considered reaction, while the leaching is minimized and the long-term catalyst life improved. This provides an opportunity for the development of active and stable Pd systems to be used under flow conditions, overcoming the limitations associated to the intrinsic mechanistic pathways of the Negishi reaction. A catch and release mechanism can be established favored by the presence of the supported ionic liquid-like phases. SILLPs with a relatively low loading of methylimidazolium units provided the most efficient systems to be used in conjunction with the immobilized NHC–Pd–RuPhos.

## Supporting Information

File 1 Experimental procedures and spectra. General flow reactions set-up.
