# Supplementary material for: Synergy between supported ionic liquid-like phases and immobilized palladium N-heterocyclic carbene–phosphine complexes for the Negishi reaction under flow conditions
Source: Beilstein J Org Chem. 2020 Aug 6;16:1924–35. doi: 10.3762/bjoc.16.159 (PMC7418103; doi:10.3762/bjoc.16.159)
Supplement: File 1 — Experimental procedures and spectra. General flow reactions set-up. [file Beilstein_J_Org_Chem-16-1924-s001.pdf]

BEILSTEIN JOURNAL OF ORGANIC CHEMISTRY

Supporting Information

for

Synergy between supported ionic liquid-like phases and immobilized palladium N-heterocyclic carbene–phosphine complexes for the Negishi reaction under flow conditions

Edgar Peris, Raúl Porcar, María Macia, Jesús Alcázar, Eduardo García-Verdugo and Santiago V. Luis

Beilstein J. Org. Chem. **2020,** *16,* 1924–1935. doi:10.3762/bjoc.16.159

Experimental procedures and spectra. General flow reactions set-up

License and Terms: This is a supporting information file under the terms of the Creative Commons Attribution License (http://creativecommons.org/licenses/by/4.0). Please note that the reuse, redistribution and reproduction in particular requires that the authors and source are credited.
The license is subject to the *Beilstein Journal of Organic Chemistry* terms and conditions: (https://www.beilstein-journals.org/bjoc)

Index:

1. Synthesis of ligands **2a**,**b** SI.2
2. Synthesis of SILLPs **3a**,**b** SI.3
3. Synthesis of Pd-NHC-SILLPs complexes (**4a**,**b**) SI.3
4. Synthesis of phosphine-Pd-NHC-SILLP complexes (**8a**,**b**) SI.4
5. Synthesis of SILLPs **9a**–**c** and **10** SI.4
6. Synthesis of Pd-SILLP **11** SI.4
7. Synthesis of PdNPs-SILLPs (**12a**,**b**) SI.4
8. General flow procedure for the Negishi cross-coupling SI.5
9. General batch procedure for the Negishi cross-coupling SI.5
10. ^{1}H-NMR from the synthesis of **7** catalyzed by **11** SI.6
11. General flow reactions set-up SI.7

1. Synthesis of ligands 2a,b. The synthesis was performed following the procedure previously described in reference 1.

1-mesityl-1H-imidazole (2a). An aqueous solution of glyoxal (30%, 6.2 mmol/mL, 0.1 mol) was added to a solution of 2,4,6-trimethylaniline (14.10 mL, 0.1 mol) in 50 mL of methanol. The mixture was stirred at rt for 24 h. A yellow precipitate was formed and then NH_4Cl (10.70 g, 0.2 mol) followed by an aqueous solution of 40% formaldehyde (10 mL, 0.2 mol) were added to this suspension. The resulting mixture was diluted with MeOH (400 mL) and heated at reflux. Then, 14 mL of aqueous 85% H_3PO_4 were added dropwise to the reaction mixture for 1 h. The reaction was refluxed for 24 h and the progress monitored by thin layer chromatography (1:1 hexane/ethyl acetate, Rf = 0.39). When the reaction was completed, the mixture was concentrated to dryness and the residue obtained added to ice water and neutralized with a solution of 40% KOH up to reach pH = 9. The resulting mixture was extracted with diethyl ether (5 × 150 mL). The organic phases were combined and washed with NaCl saturated water, dried with Na_2SO_4, concentrated to dryness and purified by flash chromatography on silica gel to afford a yellow solid.
Yield=65%. IR(cm^{-1}) ATR: 3095, 2964, 2926, 2869, 1645, 1495, 1066, 819, 810, 766, 669. ^1H NMR (400 MHz, $CDCl_3$): δ 1.98 (s, 6H, Ar-CH_3), 2.33 (s, 3H, Ar-CH_3), 6.88 (s, 1H, H_{imid}), 6.96 (s, 2H, Ar-H), 7.23 (s,1H, H_{imid}), 7.43 (s, 1H, H_{imid}). ^{13}C RMN (400 MHz, $CDCl_3$): δ 17.3 (2CH_3), 21.5 (CH_3), 120.5 (C_{imid}), 129.5 (2$C_{aromatic}$, C_{imid}), 130.0 (2$C_{aromatic}$), 136.0 ($C_{aromatic}$), 138.0 (C_{imid}), 139.3 ($C_{aromatic}$). Elemental analysis found: %C 75.9; %H 7.3; %N 14.5; calculated for $C_{12}H_{14}N_2 \cdot 0.2\, H_2O$: %C 75.9; %H 7.7; %N 14.7.

1-(2.6-diisopropylfenyl)-1H- imidazole (2b). An aqueous solution of glyoxal (30%, 6.2 mmol/mL, 0.027 mol) was added to a solution of 2,6-diisopropylaniline (5.20 mL, 0.027 mol) in 25 mL of methanol. The mixture was stirred at rt. for 24 h. A yellow precipitate was formed and then NH_4Cl (2.89 g, 0.054 mol) followed by an aqueous solution of 40% formaldehyde (4.02 mL, 0.054 mol) were added to this suspension. The resulting mixture was diluted with MeOH (85 mL) and heated at reflux. Then, 3.6 mL of aqueous 85% H_3PO_4 were added dropwisw to the reaction mixture for 1 h. The reaction was refluxed for 24 h and the progress monitored by thin layer chromatography (1:1 hexane/ethyl acetate, Rf = 0.32). When the reaction was completed, the mixture was concentrated to dryness and the residue obtained added to ice water and neutralized with a solution of 40% KOH up to reach pH = 9. Later, the resulting mixture was extracted with diethyl ether (5 × 50 mL). The organic phases were combined and washed with NaCl saturated water, dried with Na_2SO_4, concentrated to dryness and purified by flash chromatography on silica gel to afford a yellow solid.
Yield=50%. IR(cm^{-1}) ATR: 3094, 2964, 2926, 2867, 1645, 1493, 1470, 1066, 908, 809, 767,669. ^1H NMR (400 MHz, $CDCl_3$): δ 1.99 (d, 12H, -CH(CH_3)$_2$), 2.34 (m, 2H, Ar-CH(CH_3)$_2$), 6.89 (t, 2H, H_{imid}), 6.97 (s, 2H, Ar-H), 7.23 (s, 1H, H_{imid}), 7.44 (t, 1H,

Ar-H). Elemental analysis found: %C 77.6; %H 8.6; %N 11.8; calculated for $C_{15}H_{20}N_2·0.5H_2O$: %C 77.4; %H 8.9; %N 12.0.

2. Synthesis of supported ionic liquid-like phases (SILLPs) (3a,b). The synthesis was performed following the previously described procedure in reference 2.

Synthesis of polymer 3a. **2a** (4.93 g, 26.5 mmol) was dissolved in DMF (50 mL) and the Merrifield resin (7.36 g, 1.2 mmol Cl/g, 8.83 mmol) was then added. The mixture was stirred for 2 days at 80 ºC. The resulting polymer was filtered and washed with THF (3 × 15 mL), CH_2Cl_2 (3 × 15 mL) and MeOH (3 × 15 mL) and dried in a vacuum oven. IR (cm^{-1}) ATR: 3026, 2922, 1603, 1541, 1491,1451, 759, 698. Elemental analysis found: %C 83.4; %H 7.3; %N 2.1. Loading imidazolium (mmol/g): 0.75.

Synthesis of polymer 3b. Same procedure than for **3a**, but employing **2b** (2.23 g, 9.76 mmol), DMF (22 mL) and Merrifield resin (2.72 g, 1.2 mmol Cl/g, 3.25 mmol). IR (cm^{-1}) ATR: 3026, 2925, 1542, 1494, 1453, 758, 697.
Elemental analysis found: %C 81.9; %H 7.2; %N 1.8.
Loading imidazolium (mmol/g): 0.64.

3. General procedure for the synthesis of Pd-NHC-SILLP complexes (4a,b)[3]

Synthesis of supported Pd-NHC-SILLP complex 4a. The polymer **3a** (1.19 g, 0.925 mmol) was suspended in dry THF (20 mL). Then, potassium *tert*-butoxide (0.24 g, 1.85 mmol) was added and the system was stirred for 30 min. $Pd(OAc)_2$ (0.22 g, 0.925 mmol) was then added. The mixture was stirred for 2 h at 50 ºC. The resulting complex was filtered and washed with THF (3 x 15 mL), CH_2Cl_2 (3x15 mL)and MeOH (3 × 15 mL) and dried in a vacuum oven. IR (cm^{-1}) ATR: 3026, 2918, 1602, 1492, 1451, 1030, 758, 697. Elemental analysis found %C 79.2; %H 7.2; %N 1.2. Experimental Pd loading (mmol/g): 0.428.

Synthesis of supported Pd-NHC-SILLPs complex 4b
Same procedure than for **4a**, employing **3b** (1.09 g, 0.725 mmol), dry THF (20 mL), *t*-BuOK (0.195 g, 1.451 mmol), $Pd(OAc)_2$ (0.166 g, 0.725 mmol).
IR (cm^{-1}) ATR: 3025, 2923, 1600, 1490, 1451, 1182, 758, 698.
Elemental analysis found %C 80.2; %H 7.4; %N 1.3.
Experimental Pd loading (mmol/g): 0.464.

4. General procedure for the synthesis of phosphine-Pd-NHC-SILLP complexes (8a,b)

Synthesis of supported RuPhos-Pd-NHC-SILLPs complex 8a

The preparation of the NHC-Pd-RuPhos complex **8a** was carried out by suspending **4a** (1.5 g 0.642 mmol) in a solution of RuPhos (0.61 g, 1.28 mmol) in dry THF (15 mL) under stirring and at rt for 24 h. The corresponding modified immobilized NHC-Pd-RuPhos **8a** was isolated by filtration, washed with THF (3 × 15 mL), CH_2Cl_2 (3 × 15 mL) and MeOH (3 × 15 mL) to remove the non-coordinated RuPhos, and dried in a vacuum oven until constant weight. Experimental Pd loading by ICP-MS (mmol/g): 0.37.

Synthesis of supported RuPhos-Pd-NHC-SILLP complex 8b

The preparation of the NHC-Pd-RuPhos complex **8b** was carried out by suspending **4b** (0.75 g 0.348 mmol) in a solution of RuPhos (0.325 g, 0.696 mmol) in dry THF (10 mL) under stirring and at rt for 24 h. The corresponding modified immobilized NHC-Pd-RuPhos **8b** was isolated by filtration, washed with THF (3 × 15 mL), CH_2Cl_2 (3 × 15 mL) and MeOH (3 × 15 mL) to remove the non-coordinated RuPhos, and dried in a vacuum oven until constant weight. Experimental Pd loading by ICP-MS (mmol/g): 0.52 .

5. General procedure for the synthesis of Supported Ionic Liquid-like Phases (SILLPs) (9a–c and 10)

The polymers **9a-c** (**9a** 1.01 mmol of IL-like units /g, **9b** 0.97 mmol of IL-like units /g and **9c** 0.88 mmol of IL-like units /g) and **10** (3.79 mmol of IL-like units /g) were obtained following the experimental procedures previously described by our group in references 2 and 3.

6. Procedure for the synthesis of Pd-SILLP 11

SILLP **10** (1 g, 3.79 mmol of IL-like units /g) was introduced in a solution of $PdCl_2$ (1000 ppm $PdCl_2$ in 1% HCl in miliQ® H_2O, 100 mL). The mixture was stirred (350 rpm) using an orbitalic stirrer at rt for 5 h. The colour of the orange solution was completely absorbed by the SILLP beads indicating full Pd absorption. The resulting supported complex was filtered, washed with miliQ H_2O (3 × 20 mL) and dried under vacuum until constant weight. Experimental IL-like units loading (mmol/g): 3.79. Experimental Pd loading (mmol/g): 0.56.

7. Procedure for the synthesis of PdNPs-SILLPs (12a-b)

Synthesis of PdNPs-SILLP 12a. $NaBH_4$ (0.2 g) was dissolved in 1:4 EtOH/H_2O (12 mL) and of Pd-SILLP **11 (**250 mg) was added to the solution. The mixture was stirred at rt for 3 h. After this time, the resin was filtered, washed with 1:4

EtOH/H_2O (3x20 mL) and dried under vacuum until constant weight. IL-like units loading (mmol/g): 3.79. Pd loading (mmol/g) calculated by ICP-MS: 0.56.

Synthesis of PdNPs-SILLP 11b. Pd-SILLP **11** (250 mg) and EtOH (4 mL) were introduced in a Microwave vial. The mixture was heated by Microwave for 2 h (200 °C, 300 psi, 120 W). The resulting resin was filtered, washed with EtOH (3x20 mL) and dried under vacuum until constant weight. IL-like units loading (mmol/g): 3.79. Pd loading (mmol/g): 0.56.

8. General flow procedure for the Negishi cross-coupling: A solution of benzylzinc bromide (**5**) (0.2 M in dry THF) was mixed with a solution of methyl 4-bromobenzoate (**6**) (0.1 M in dry THF) in a T piece to achieve the desired flow rate of 0.1 mL/min. The resulting mixed solution was passed through a 6.6 mm internal diameter Omni-fit® column at 60 °C containing the immobilised catalyst and the scavenger SILLPs (weight ratio 1:3), depending of the experimental conditions. The solution was collected at the outlet of the reactor at different time intervals. The crude of the reaction was quenched with a saturated solution of ammonium chloride and extracted with AcOEt. The organic layer was separated, dried (MgSO4) and filtered. Yields were calculated from the reaction crude by GC and confirmed by ^{1}H NMR.

9. General batch procedure for the Negishi cross-coupling: Methyl 4-bromobenzoate (**6**) (54 mg, 0.25 mmol), the Pd catalyst (5 mol %, 0.0125 mmol) and the scavenger SILLP when required (weight ratio 1:3, Pd cat : scavenger) were introduced in a sealable vial. The vial was sealed and purged with N_2. A solution of benzylzinc bromide (**5**) (0.5 M in dry THF, 1 mL, 0.5 mmol of **5**) was injected through the vial septum to complete a total solvent volume of 2 mL. The mixture was stirred at 60 °C. Samples were collected at different times using a syringe attached to a microfilter in order to avoid collecting catalyst solid particles. The crude of the reaction was quenched with a saturated solution of ammonium chloride and extracted with AcOEt. The organic layer was separated, dried ($MgSO_4$) and filtered. Yields were calculated from the reaction crude by GC and confirmed by ^{1}H-NMR.

CG method: Column BP20, Injector: 230 °C, Oven: 60 °C (step 1), 60-250 °C (35 °C/min, step 2), 250 °C (60 min, step 3), Pressure: 15.00 Psi, Detector: 300 °C, Helium flow: 25 mL/min, Hydrogen flow: 30 mL/min, Air flow: 300 mL/min, t_R = 12.22 min

10. ^{1}H-NMR obtained from the reaction crude in the synthesis of methyl 4-benzylbenzoate (7) by reaction of 1 eq. of methyl 4-bromobenzoate and 2 eq. of benzylzinc bromide catalyzed by 11.

Yield was calculated by GC, but the integration of the main peaks of the ^{1}H-NMR data showed a conversion > 99 % and yield of 99%

Conversion (%) = [1- (a /a*)] x 100

Yield (%) = { (a/3) / [(a/3) + (a*/3)] } x 100

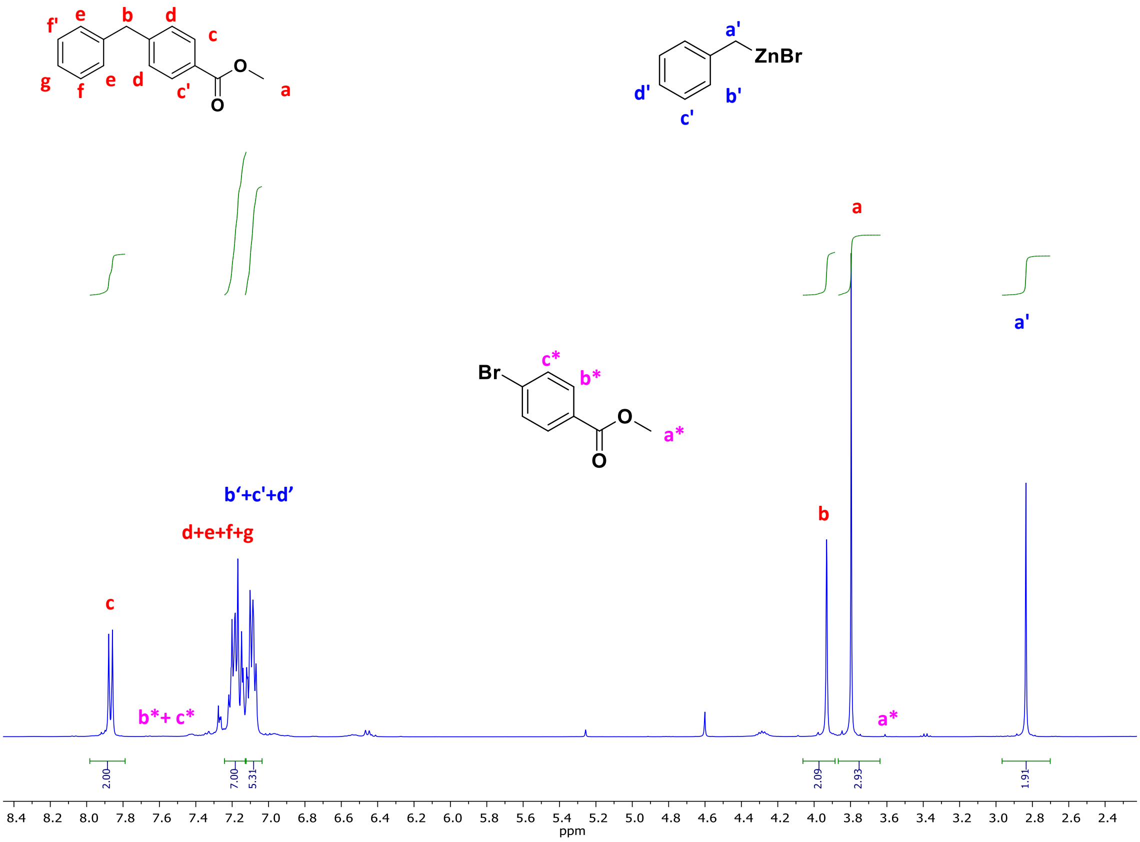

^{1}H NMR **7** (300 MHz, $CDCl_3$) δ 7.96 (d, 2H), 7.29-7.17 (m, 7H), 4.02 (s, 2H), 3.89 (s, 3H)

11. General flow reactions set-up.

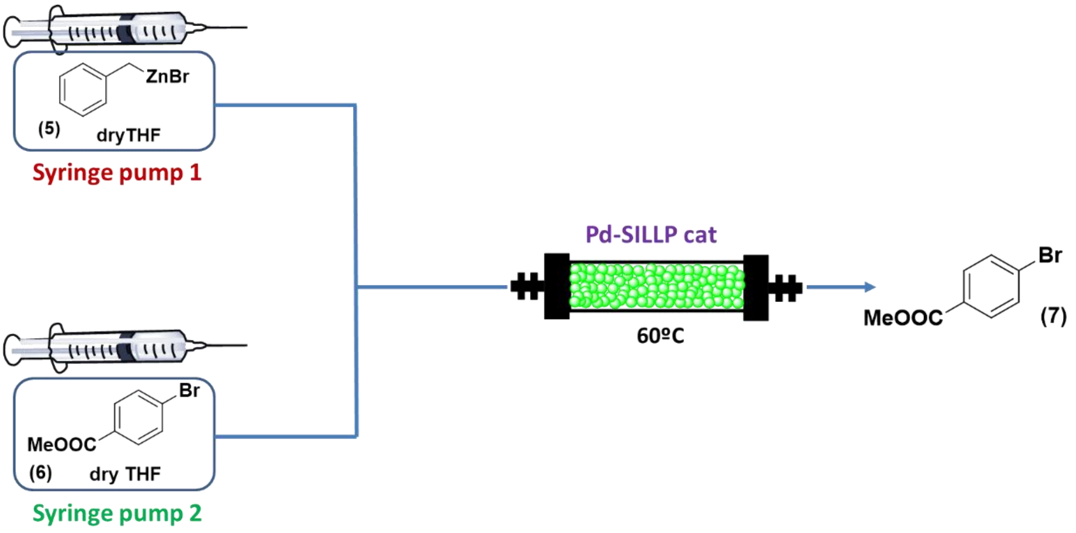

General scheme for the continuous flow used in the Negishi cross-coupling reaction studied.

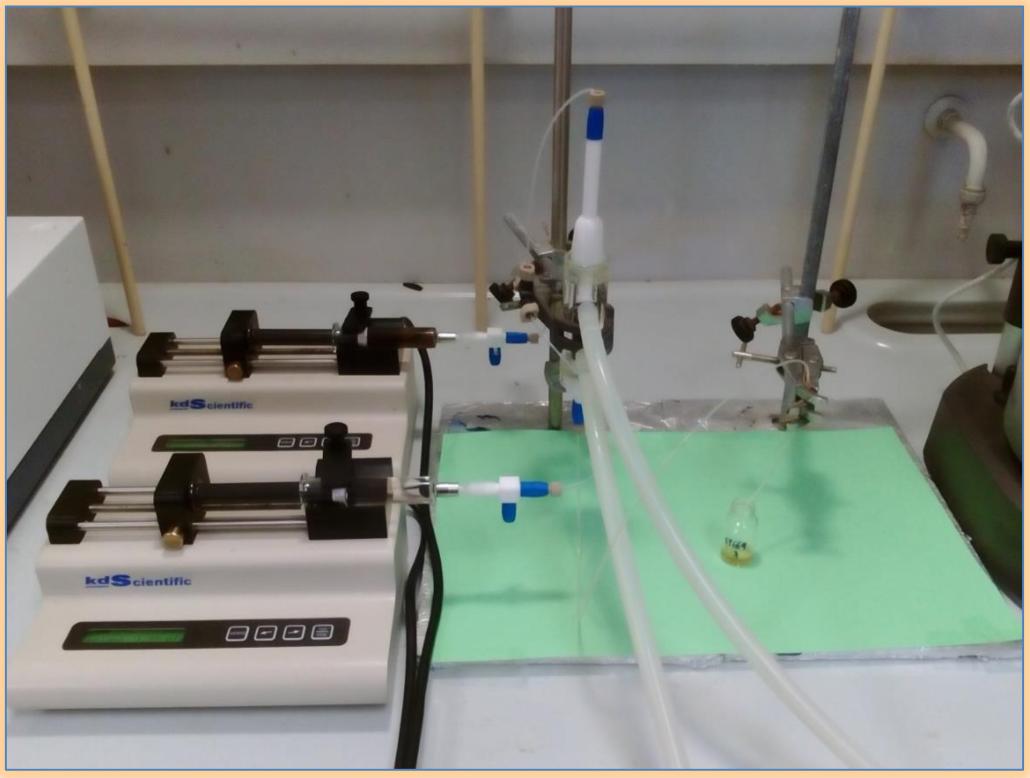

General set-up used to perform the Negishi cross-coupling reaction between benzylzinc bromide (**5**) and methyl 4-bromobenzoate (**6**). Syringe pumps from kdScientific and Hamilton 10 mL syringes were used. Glass Omnifit® columns 006RG-10-10 (0.7854 cm diameter × 10 cm length) were used as fixed-bed reactors. The reactor was heated at 60 ºC by iPrOH reflux.

References.

1 *Design and synthesis of new gold Palladium and Rhodium complex with chiral ligands base on dioxalane and proton sponge backbone. Study of their heterogeneization and catalytic behavior*. Villaverde, G., Thesis Doctoral, Universidad Autónoma de Madrid, 2011, https://repositorio.uam.es/bitstream/handle/10486/7768/43068_villaverde_cantizano_gonzalo.pdf

2 Burguete, M. I.; Erythropel, H.; García-Verdugo, E.; Luis, S. V.; Sans, V. *Green Chem*. **2008**, *10*, 401-407.

3 Sans, V.; Gelat, F.; Burguete, M. I.; García-Verdugo, E.; Luis, S. V. *Cat. Today*, **2012**, 196, 137-147.
